# Artificial Dermis for Protecting Gastrointestinal Mucosal Defects: A Feasibility Study in ex vivo and in vivo Porcine Models

**DOI:** 10.1055/a-2953-8822

**Published:** 2026-09-14

**Authors:** Tatsunori Minamide, Hiroaki Ikematsu, Shigeki Morita

**Affiliations:** 1 Department of Gastroenterology 89275 IMSUT Hospital, The University of TokyoTokyoTokyoJapan; 2 Department of Pathology 89275 The Institute of Medical Science, The University of TokyoTokyoTokyoJapan

**Keywords:** endoscopy upper GI tract, endoscopic resection (ESD, EMRc, ...), ulcers (peptic and other), quality and logistical aspects, performance and complications

## Abstract

Although post-endoscopic submucosal dissection (ESD) mucosal defects can lead to delayed bleeding and perforation, an optimal protective method has not yet been established. This study aimed to evaluate the feasibility of using artificial dermis to protect against gastrointestinal mucosal defects. ex vivo and in vivo porcine models were used. Artificial dermis sheets (collagen monolayer or collagen–silicone bilayer) were applied to 2-cm post-ESD ulcers in the stomach, rectum, and esophagus and fixed using standard endoscopic clips. Fixation success, coverage durability, adverse events, and histopathologic findings were evaluated. In the ex vivo experiments, artificial dermis was successfully fixed with clips, both manually and endoscopically, without material damage (4/4 sites per experiment). In the in vivo experiments, fixation was successful at all planned gastric and esophageal sites (5/5, 100%). The bilayer sheets appeared to remain in place more consistently at day 3 in the stomach. No serious adverse events were observed. Endoscopic application of artificial dermis was technically feasible for temporary coverage of gastrointestinal mucosal defects in porcine models, with collagen–silicone bilayer sheets showing more consistent retention during the initial observation period. However, further clinical studies are warranted.

## Introduction

Endoscopic resection, including endoscopic submucosal dissection (ESD) for gastrointestinal tumors, is widely adopted as a minimally invasive and highly curative treatment modality. 1 However, serious complications associated with post-ESD ulcers, such as postoperative bleeding and delayed perforation, remain problematic. The incidence of delayed bleeding in the stomach is generally approximately 5% 2 but increases substantially to approximately 30% in high-risk patients, including those taking antithrombotic drugs. 3 Although the incidence of delayed perforation is low (approximately 0.4%), it is a serious complication that often requires emergency surgery. 2 To prevent these complications, several methods including clip-based suturing techniques, 4 endoscopic hand suturing, 5 and polyglycolic acid (PGA) sheets 6 7 have been developed to protect against mucosal defects. However, these approaches are often limited by technical difficulty for extensive ulcers, cumbersome procedures, and high costs. Therefore, a simple biomaterial that can be delivered endoscopically and provide temporary coverage of post-ESD ulcers may be an attractive alternative.

An artificial dermis (Terudermis; GC Corporation, Tokyo, Japan) consists of a 3-mm atelocollagen sponge with or without a 0.2-mm silicone layer ( Fig. 1 ). When applied to mucosal or skin defects, the collagen layer facilitates dermis-like tissue formation via cellular infiltration from the wound bed. This material has been successfully used to repair severe skin and mucosal defects caused by burns, trauma, surgical wounds, and cleft palate surgery and has demonstrated favorable biocompatibility, tissue regeneration, and acceptable long-term safety profiles in clinical practice. 8 9 During endoscopy, the material retains flexibility and sufficient thickness by absorbing liquids, offering excellent maneuverability, stable adhesion, and a high protective potential. Based on these characteristics, we hypothesized that artificial dermis could serve as a temporary shielding material for gastrointestinal mucosal defects.

**Fig. 1:**
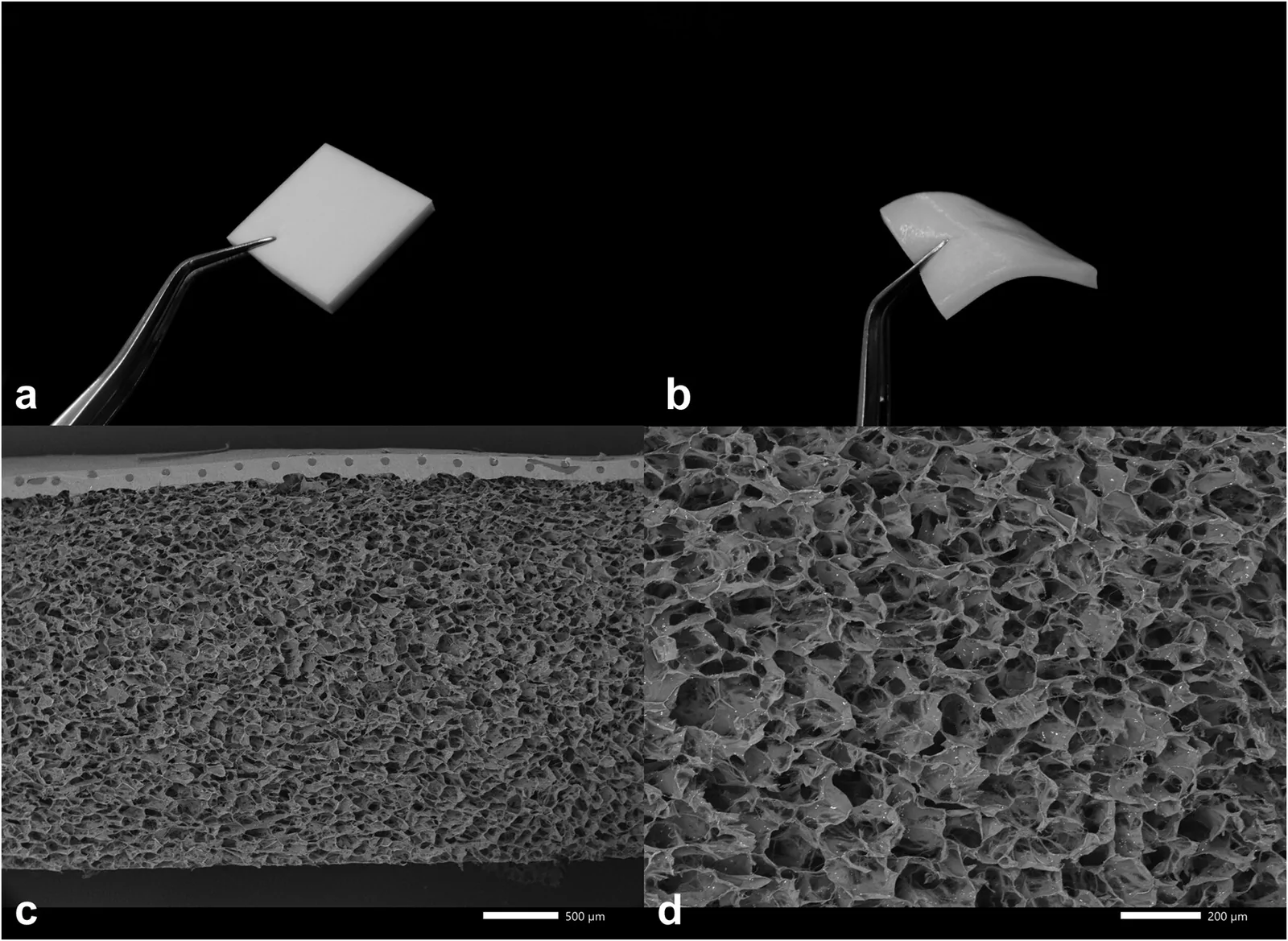
Artificial dermis. ( **a** ) Artificial dermis in a dry state. ( **b** ) Artificial dermis after soaking in water. It becomes soft and deformable. ( **c** ) Microscopic cross-sectional image of a collagen–silicone bilayer sheet. The upper thin layer consists of silicone, while the lower thick layer consists of collagen. The scale bar corresponds to 500 μm. ( **d** ) A highly magnified microscopic image of the collagen layer. It features a scaffold-like structure containing numerous fine spaces, making it highly absorbent. The scale bar corresponds to 200 μm.

In this study, we evaluated the feasibility of using artificial dermis to protect against gastrointestinal mucosal defects in ex vivo and in vivo animal models.

## Material and Methods

### Ex Vivo Experiments

We obtained pig stomachs and recta that were removed during slaughter for food purposes. Two board-certified endoscopists performed ESD according to the standard procedure, including marking, local saline injection into the submucosal layer, circumferential mucosal incision, and submucosal dissection, to create 2-cm artificial ulcers. These procedures were performed manually in the first experiment and endoscopically in the second experiment using a single-channel upper gastrointestinal endoscope (GIF-H260; Olympus, Tokyo, Japan). An electrosurgical knife (DualKnife J, KD-655Q; Olympus) and electrosurgical unit (VIO300D; ERBE, Tübingen, Germany) were also used. After soaking in water to soften the material, a 2.5-cm artificial dermis sheet (Terudermis, TD-A006N or TD-M006S; GC Corporation) ( Fig. 1 ) was placed over each ESD ulcer and fixed using endoscopic clips (EZ Clip, HX-610-135 or HX-610-135L; Olympus and Resolution Clip, M00522600; Boston Scientific, Massachusetts, United States). Two types of sheets were used: a collagen monolayer and a collagen–silicone bilayer.

In the first experiment, we investigated whether artificial dermis could be applied without damage using endoscopic clips in opened porcine organs. Artificial dermis sheets were manually placed over four ulcers (three gastric and one rectal) and fixed with clips. In the second experiment, we evaluated whether the artificial dermis could be applied using an endoscope inside unopened porcine organs following ESD. For four ulcers (three gastric and one rectal), artificial dermis sheets were delivered to the mucosal defects using grasping forceps, positioned over the defects, and fixed with endoscopic clips.

The ex vivo experiment was conducted as a preliminary assessment of technical feasibility. The endpoint was technical success, defined as successful deployment and clip fixation of the artificial dermis at the intended site without material damage. Progression to the in vivo experiment was based on successful deployment and fixation without material damage in the ex vivo setting.

### In Vivo Experiments

The animal experiment was approved by the Animal Experiment Committee of the Institute of Medical Science, University of Tokyo (approval number: 2025IMS137; approval date: September 17, 2025).

A female pig weighing approximately 40 kg was used. Two board-certified endoscopists performed ESD using a single-channel upper gastrointestinal endoscope (GIF-Q260J; Olympus), an electrosurgical knife (ITknife 2 or DualKnife J; Olympus), and an electrosurgical unit (VIO300D; ERBE). ESD was performed under general anesthesia, as described earlier, to create artificial ulcers measuring 2 cm in diameter at eight gastric sites (four in the body and four in the antrum) and two esophageal sites. After softening in water, a 2.5-cm artificial dermis sheet (TD-A006N or TD-M006S; GC Corporation) was placed over each ESD ulcer using grasping forceps and fixed with endoscopic clips (EZ Clip, HX-610-135 or HX-610-135L; Olympus). Similar to the ex vivo experiments, two types of sheets were used. Four gastric body or antrum ulcers were assigned to the following conditions: collagen–silicone bilayer sheet with clip fixation, collagen monolayer sheet with clip fixation, collagen monolayer sheet without clip fixation, and uncovered control. Uncovered lesions served as controls for natural healing, whereas lesions covered without clip fixation were included to assess spontaneous adherence of the material. In the esophagus, one ulcer was left uncovered, while the other had a collagen monolayer sheet clipped in place. Two clips were used per gastric ulcer, and four clips were used per esophageal ulcer.

The pig was gradually given water and feed starting the day after the procedure and was given normal solid feed one week later. Vonoprazan fumarate (40 mg) was administered orally once daily and mixed with the feed. Follow-up endoscopy was performed at 3, 7, and 14 days after the procedure. The pig was sacrificed after the 14-day examination. Resected stomach and esophagus were fixed in formalin and embedded in paraffin, and tissue specimens were prepared from each ESD site. Hematoxylin and eosin staining was performed, followed by blinded qualitative evaluation by an expert pathologist focusing primarily on inflammatory cell infiltration, ulcer healing, and foreign-body reaction. No formal semiquantitative scoring system was applied because of the exploratory nature of this pilot study.

The in vivo experiment was conducted as an exploratory assessment of endoscopic applicability and short-term retention. The primary endpoint was technical success of artificial dermis deployment and fixation. Secondary endpoints included procedure time, coverage durability, adverse events, and histopathologic findings. Procedure time was defined as the interval from grasping artificial dermis with the forceps to completion of clip fixation on the target mucosal defect. Coverage durability was defined as complete retention when the sheet remained intact and adhered to the same site; partial retention when the sheet partially disappeared but remained adhered to the same site; and lost in all other cases.

## Results

In the first ex vivo experiment, manual clip fixation of the artificial dermis was successfully achieved at all four sites without material damage (100%) ( Fig. 2a–c ). The material did not tear when repeatedly moved manually. In the second ex vivo experiment, endoscopic delivery and clip fixation were successful at all four sites (100%), enabling complete coverage of the post-ESD ulcers ( Fig. 2d–g ).

**Fig. 2:**
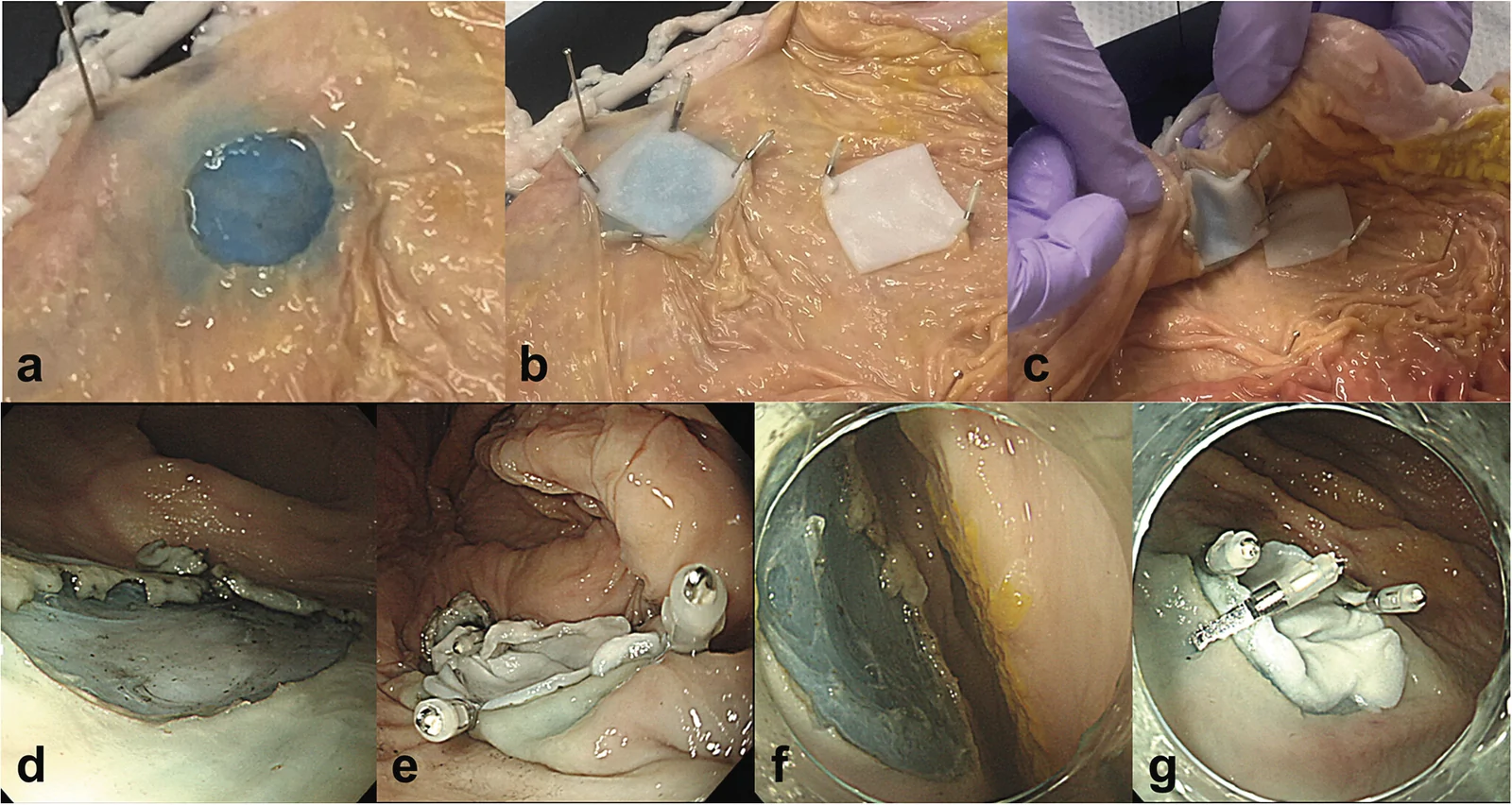
Ex vivo porcine experiments. ( **a–c** ) Endoscopic images of the first ex vivo experiment in the opened porcine organs. ( **a** ) Gastric ulcers after endoscopic submucosal dissection. ( **b** ) Gastric ulcers covered with artificial dermis sheets using clips without material damage. ( **c** ) Artificial dermis did not tear when manually moved repeatedly. ( **d–g** ) Endoscopic images of the second ex vivo experiment inside unopened porcine organs. ( **d, e** ) Gastric antrum ulcer before ( **d** ) and after ( **e** ) covering with an artificial dermis using clips. ( **f, g** ) Gastric antral ulcer before ( **f** ) and after ( **g** ) coverage with artificial dermis using clips.

In the in vivo experiments, clip fixation was successfully achieved at all five planned sites (100%; four gastric and one esophageal) ( Fig. 3 , Table 1 ). The median procedure time for completion of fixation was 5 min (range 2–7 min). At the 3-day follow-up, complete retention was confirmed for one collagen–silicone bilayer sheet in the stomach, while partial retention was confirmed for one collagen–silicone sheet in the stomach and one collagen sheet in the esophagus ( Fig. 4 , Table 1 ). All remaining sheets were lost. Although one clip detached from the silicone sheet fixed in the antrum, thick white deposits were observed at the ulcer base, suggesting residual collagen. Regarding the collagen monolayer sheet in the esophagus, thick white deposits were observed on both the clip base and ulcer base, which might be residual collagen. At the 7-day follow-up, all three sheets that had shown complete or partial retention were lost ( Fig. 5a,c,e ). All the ulcers healed and were scarred after 14-day follow-up ( Fig. 5b,d,f ).

**Fig. 3:**
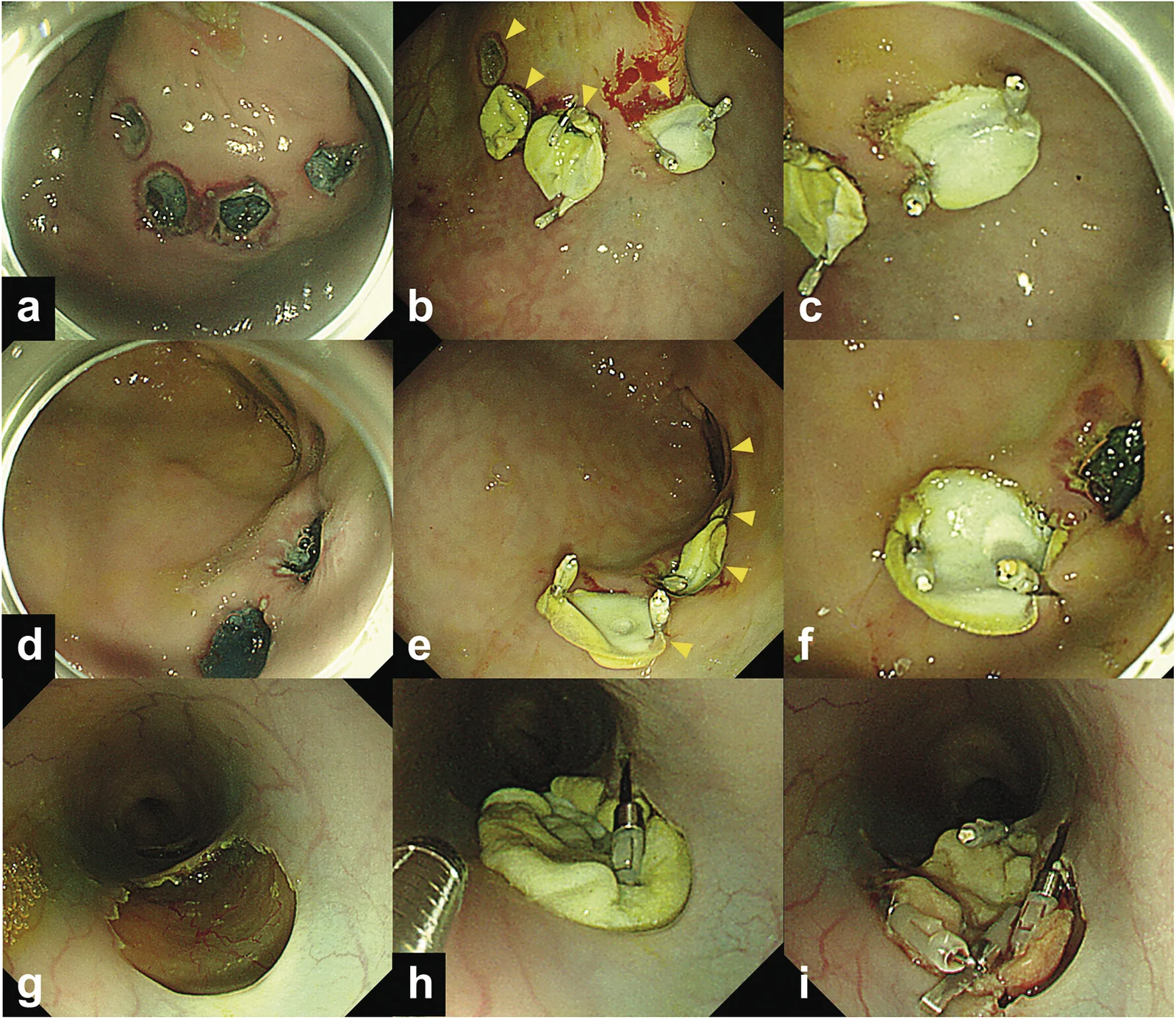
In vivo porcine experiments on the day of endoscopic submucosal dissection. ( **a–c** ) Endoscopic images of the gastric body. ( **a** ) Gastric body ulcers after endoscopic submucosal dissection (ESD). ( **b** ) Four gastric body ulcers (arrowheads) were assigned to different treatment conditions. From right to left: collagen–silicone bilayer sheet with clip fixation, collagen monolayer sheet with clip fixation, artificial dermis without clip fixation, and uncovered control. ( **c** ) Gastric body ulcer covered with a collagen–silicone bilayer sheet. ( **d–f** ) Endoscopic images of the gastric antrum. ( **d** ) Gastric antrum ulcers after ESD. ( **e** ) Four gastric antrum ulcers (arrowheads) were assigned to the same treatment conditions as the gastric body. From proximal to distal: collagen–silicone bilayer sheet with clip fixation, collagen monolayer sheet with clip fixation, artificial dermis without clip fixation, and uncovered control. ( **f** ) Gastric antrum ulcer covered with a collagen–silicone bilayer sheet. ( **g–i** ) Endoscopic images of the esophagus. ( **g** ) Esophageal ulcers after ESD. ( **h** ) Esophageal ulcer covered with a collagen monolayer sheet before clip fixation. ( **i** ) Esophageal ulcer covered with a collagen monolayer sheet using clips.

**Table 1:** Technical success and coverage durability at follow-up according to treatment assignments.

Treatment assignments	Technical success	Follow-up at day 3	Follow-up at day 7
Gastric body			
Bilayer sheet + clips	Yes	Complete retention	Lost
Monolayer sheet + clips	Yes	Lost	Lost
Monolayer sheet alone	―	Lost	Lost
No coverage	―	―	―
Gastric antrum			
Bilayer sheet + clips	Yes	Partial retention	Lost
Monolayer sheet + clips	Yes	Lost	Lost
Monolayer sheet alone	―	Lost	Lost
No coverage	―	―	―
Esophagus			
Monolayer sheet + clips	Yes	Partial retention	Lost
No coverage	―	―	―

Technical success was defined as successful deployment and clip fixation of the artificial dermis at the intended site without material damage. Coverage durability was defined as complete retention when the sheet remained intact and adhered to the same site; partial retention when the sheet partially disappeared but remained adhered to the same site; and lost in all other cases.

**Fig. 4:**
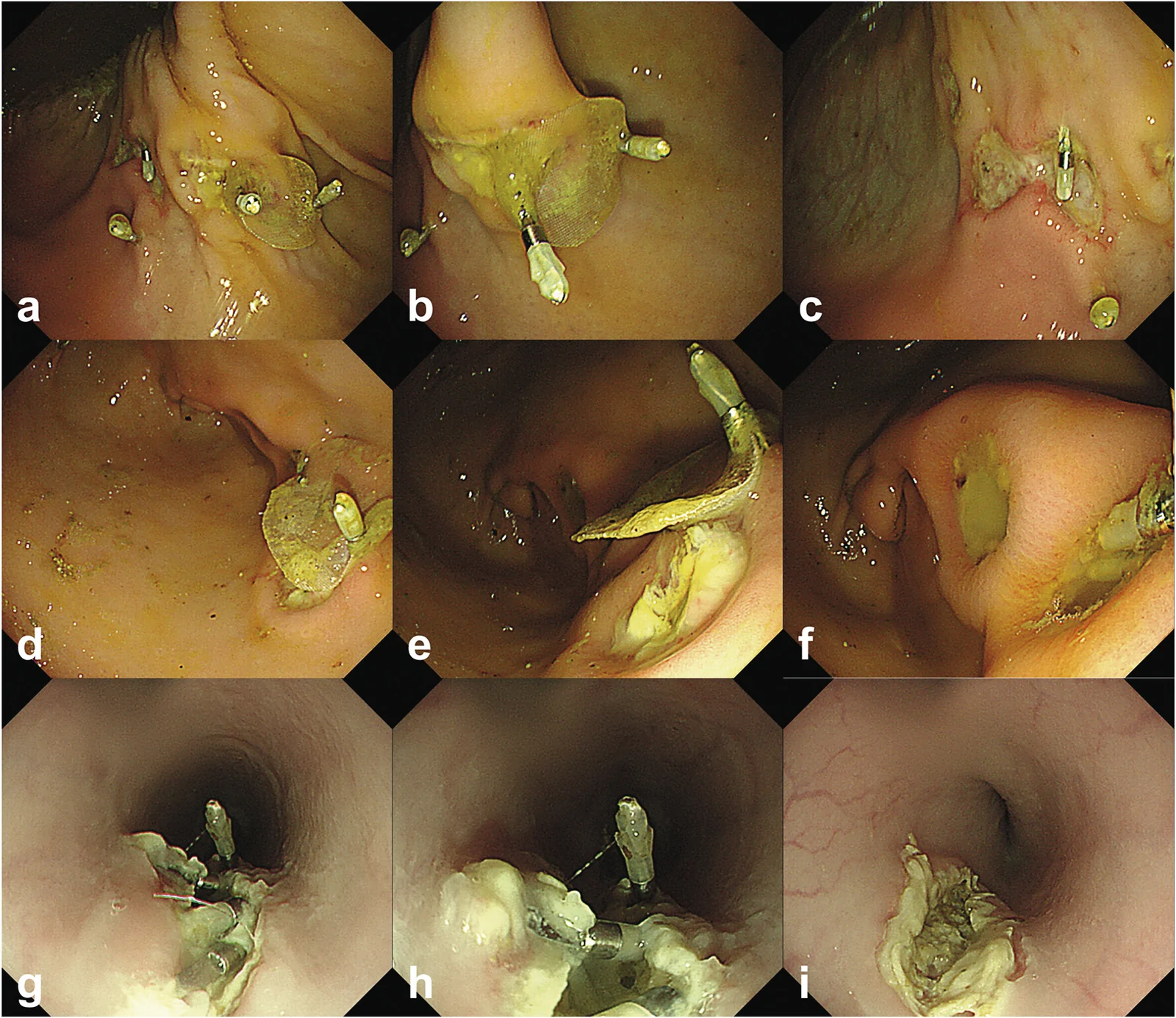
In vivo porcine experiments 3 days after endoscopic submucosal dissection. ( **a–c** ) Endoscopic images of the gastric body. ( **a** ) Distant view of gastric body ulcers following endoscopic submucosal dissection (ESD). ( **b** ) Gastric body ulcer covered with a collagen–silicone bilayer sheet. ( **c** ) Other gastric body ulcers not covered with a collagen–silicone bilayer sheet. ( **d–f** ) Endoscopic images of the gastric antrum. ( **d** ) Distant view of gastric antrum ulcers after ESD. ( **e** ) Gastric antrum ulcer covered with a collagen–silicone bilayer sheet. ( **f** ) Other gastric antrum ulcers not covered with a collagen–silicone bilayer sheet. ( **g–i** ) Endoscopic images of the esophagus. ( **g** ) Esophageal ulcer covered with a collagen monolayer sheet. ( **h** ) Close-up view of esophageal ulcer covered with a collagen monolayer sheet. ( **i** ) Esophageal ulcer left uncovered.

**Fig. 5:**
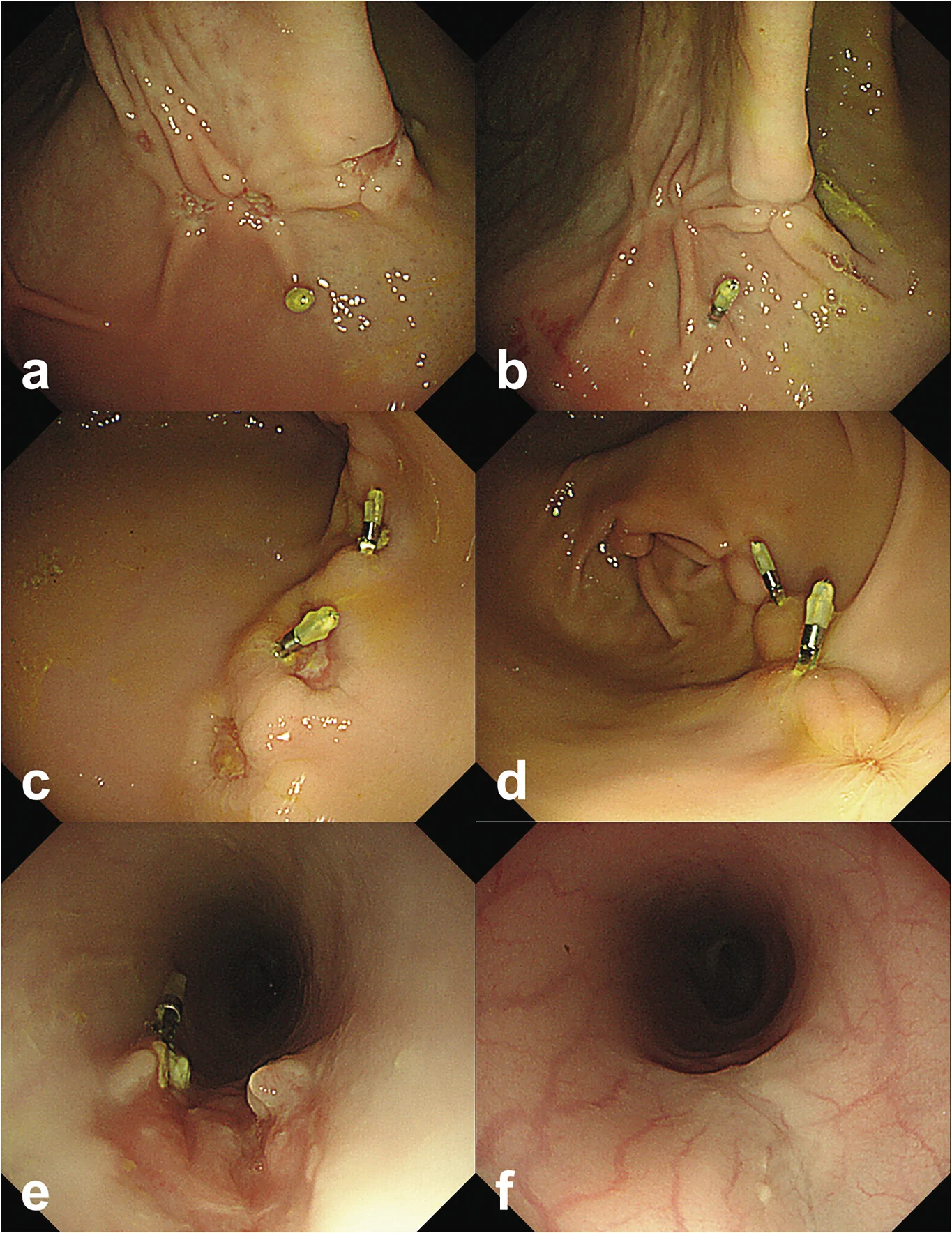
In vivo porcine experiments 7 and 14 days after endoscopic submucosal dissection. ( **a, b** ) Gastric body ulcers 7 ( **a** ) and 14 days ( **b** ) after endoscopic submucosal dissection (ESD). ( **c, d** ) Gastric antrum ulcers 7 ( **c** ) and 14 days ( **d** ) after ESD. ( **e, f** ) Esophageal ulcers covered with a collagen monolayer sheet 7 ( **e** ) and 14 days ( **f** ) after ESD.

No serious adverse events, including delayed bleeding, perforation, or gastrointestinal obstruction, occurred during the observation period. Histopathologically, no significant differences in inflammatory findings were observed between sites with and without artificial dermis fixation, suggesting a normal healing process ( Fig. 6 ).

**Fig. 6:**
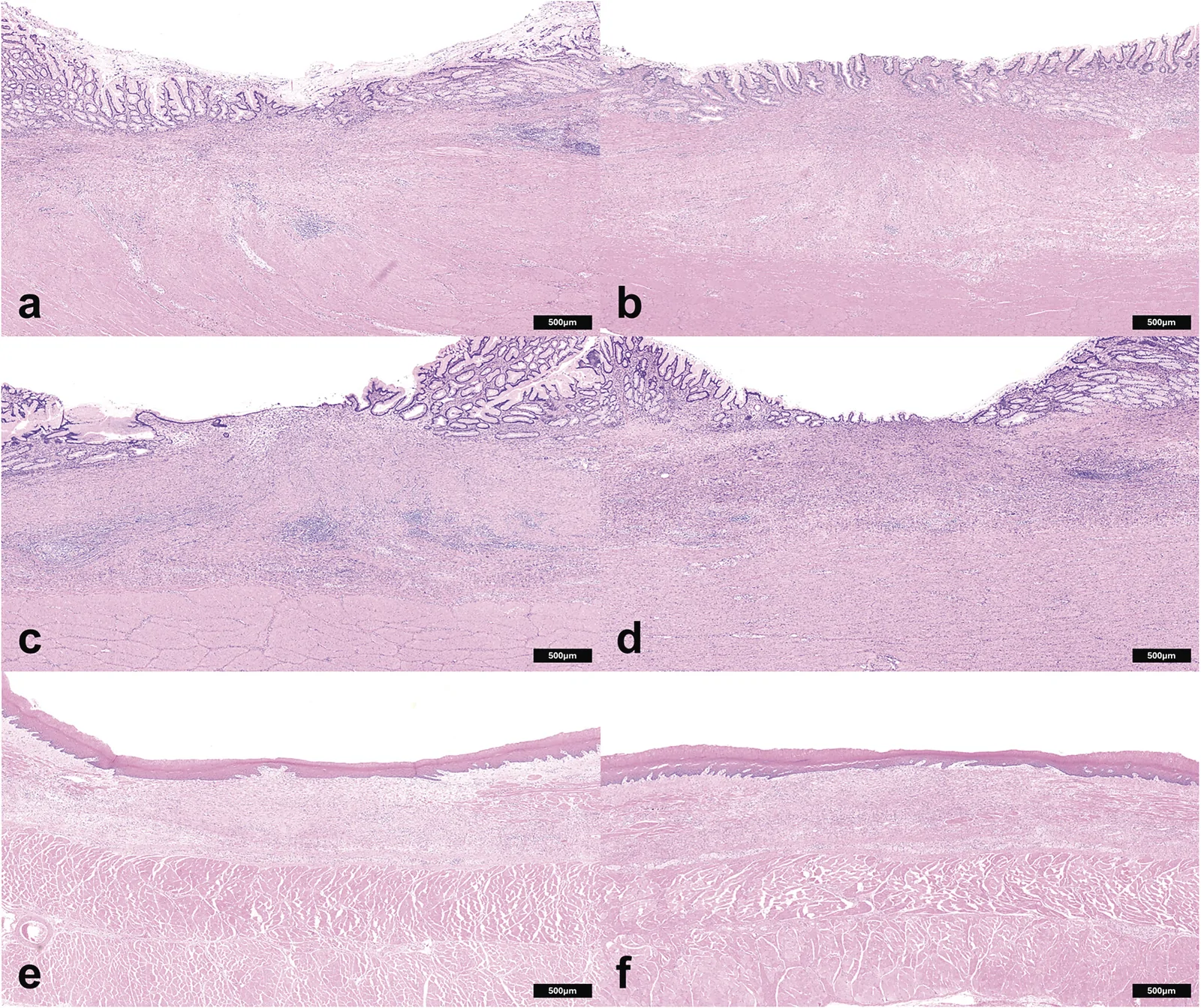
Histopathologic images of ulcer scars following endoscopic submucosal dissection by hematoxylin and eosin staining. ( **a** ) Gastric body ulcer scar covered with a collagen–silicone bilayer sheet. ( **b** ) Gastric body ulcer scar not covered by artificial dermis. ( **c** ) Gastric antrum ulcer scar covered with a collagen–silicone bilayer sheet. ( **d** ) Gastric antrum ulcer scar not covered by artificial dermis. ( **e** ) Esophageal ulcer scar covered with a collagen monolayer sheet. ( **f** ) Esophageal ulcer scar not covered by artificial dermis. The scale bars correspond to 500 μm.

## Discussion

This exploratory study demonstrated the feasibility of an endoscopic protective method using artificial dermis in ex vivo and in vivo porcine models. Unlike complete closure techniques, artificial dermis is intended to provide temporary shielding of post-ESD ulcers when complete closure is technically difficult or impractical. This technique requires only artificial dermis and standard endoscopic clips, and fixation was technically successful at all sites within a median of 5 min, suggesting a low procedural burden. No adverse effects on ulcer healing or survival were observed.

To the best of our knowledge, this is the first report of the application of artificial dermis to protect against gastrointestinal mucosal defects. Artificial dermis offers several advantages. First, its shape and size can be adjusted freely, and this biomaterial absorbs liquids and becomes soft and deformable. This allows for a more reliable application to areas of various shapes and even large defects. Second, it facilitates endoscopic handling. It is thicker than PGA, and the collagen–silicone bilayer type is considered easier to deliver and fix. It was possible to fix the sheets without damaging them using only standard clips. Third, it has a relatively low cost (approximately 452 JPY per cm ^2^ ).

We evaluated both a collagen monolayer sheet and a collagen–silicone bilayer sheet in the stomach. Artificial dermis without clip fixation was rapidly lost from all sites, suggesting that mechanical fixation is required for reliable retention. The bilayer sheet appeared to have greater durability during the early period after gastric ESD when fixed with clips. This is likely because the thin silicone layer reinforced with polyester mesh reduced tearing after clip fixation. Prolonged preservation of the underlying collagen layer may provide more robust protection against mucosal defects. Although all sheets detached by day 7, temporary retention during the early post-ESD period may still be clinically relevant because delayed bleeding frequently occurs within the first several days after ESD. 10 In contrast, although the collagen monolayer sheet appeared to persist longer in the esophagus, it may have detached or dissolved in the stomach. This difference may be attributable to organ-specific luminal environments, as the stomach is exposed to gastric acid and greater mechanical stress than the esophagus. Given the highly acidic gastric environment, long-term persistence of a collagen monolayer is theoretically unlikely. Only the collagen monolayer sheet was evaluated in the esophagus because prolonged retention was considered more likely in the absence of gastric acid exposure.

Collagen sheets dissolve in digestive fluids, including gastric acid, and are thus expected to be safe within the digestive tract. No serious adverse events were observed during the in vivo porcine experiment. However, the collagen–silicone bilayer sheet contains nonabsorbable silicone, raising concerns about local inflammation from residual material and gastrointestinal obstruction after detachment. In this study, histopathologic evaluation of the resected specimens revealed no excessive inflammation or symptoms of gastrointestinal obstruction. While these issues warrant greater caution with larger sheet sizes, using multiple sheets of a limited size or removing them after a certain period may mitigate these risks.

Before clinical application, several issues remain to be addressed. The sheet size, fixation technique, and duration of retention should be optimized to maximize protection while maintaining safety. In addition, this approach should be evaluated in larger post-ESD defects and in patients at high risk of delayed bleeding or perforation, who would benefit most from temporary ulcer shielding. Ultimately, prospective clinical studies are warranted to confirm the safety, feasibility, and efficacy of this technique.

This study had some limitations. First, it was a nonclinical investigation with a small sample size. Second, only relatively small post-ESD ulcers measuring 2 cm were evaluated. Third, the experiment was performed by two endoscopy specialists.

In conclusion, artificial dermis was feasibly applied endoscopically to protect against gastrointestinal mucosal defects in porcine models, and the collagen–silicone bilayer sheet appeared to remain in place more consistently during the initial observation period. Therefore, further clinical studies are warranted.
